# Spavin1Paper read before the meeting at Plymouth, England.

**Published:** 1899-10

**Authors:** 


### SELECTION.

### SPAVIN.[1]

By Vet.-Major Smith, A.V.D.

I have written on the subject of spavin with some degree of fulness within the last few years, and have nothing to add to my original communication.[2] All I will attempt to do here is to draw attention to the principal features connected with this disease, in order to direct the lines on which the subject could best be discussed before this representative assembly, so that we may obtain the views of the profession on each of the leading features connected with this serious form of lameness.

Is there any particular shape of hock more liable to the disease than any other?

Writer's view: I am far from clear on the subject. It is very remarkable how the most unpromising-looking hock will stand work; but if I have any prejudice it is that the short, compressed hock is more likely to develop trouble than a large well-made one, though, unfortunately, many of the latter also give way.

What are the causes of spavin?

Writer's view: (*a*) The disease begins in the bone and extends to the cartilage. Therefore, the causes operating should mainly be of the nature of shock (concussion). This concussion can be imparted when the limb is off the ground, as in extreme flexion of the joint, or, more commonly, when it is on the ground, either at the moment it receives the weight, or, perhaps more frequently, at the final propulsion as the toe is leaving the ground. (*b*) Hereditary predisposition.

What is the definition of spavin?

Writer's view: A disease affecting some of the small bones and articulations of the hock, most commonly those situated at the inner and anterior part of the joint, which shows itself usually by an enlargement of the part, though this is sometimes absent. As a matter of fact, a spavin is a very difficult thing to define. Ulceration of the joint may exist between the calcis and astragalus —a part out of sight and touch—and, therefore, no external manifestation but lameness; or the cuboid may be affected, in which

[1] Paper read before the meeting at Plymouth, England.
[2] The Journal of Comparative Pathology and Therapeutics, 1892, vol. v., No. 3.

case the enlargement is on the outer and not the inner aspect of the joint; in fact, there are many varieties of trouble, the pathological features of which are identical with spavin, and yet are scarcely covered by the definition of the disease which I have offered.

Is there more than one variety of spavin, or are there two distinct diseases of the hock which we speak of by this name? Writer's view: There are certainly two varieties of spavin—a curable and incurable form, viz.: (*a*) A variety consisting of ankylosis and exostosis, which is curable, and another (*b*) consisting of ulceration of the articular surface, with little or no ankylosis, and usually little or no exostosis, which is at present incurable. My experience leads me to think that there are two distinct diseases of the hock—*a* and *b*; both are produced by the same cause, but are totally different in character. I do not think one runs into the other, though sometimes they are combined in the same joint.

There is, however, another side to the question: The regions affected by *a* and *b* are not the same; while *a* favors the intercuneiform region, *b* prefers the extra-cuneiform, viz., the astragalus and head of the large metatarsal. It may, therefore, be that the diseases are identical, but that the region affected determines whether the lesion shall be of a constructive or destructive character. But every spavin is articular. No ankylosis can occur of any of the small bones of the hock without the articular surfaces being affected, but every articular lesion does not lead to caries. In variety *a* the articular lesion is constructive; in variety *b* destructive. The appearance presented by the articular surfaces in these two conditions is very different.

Which bones are most commonly affected in spavin?

Writer's view: Without doubt the magnum and medium; the parvum and cuboid may be attacked, the parvum more frequently than the cuboid, but very rarely are the heads of the small metatarsal bones affected. Under disease, the magnum and medium are rarely united over the whole of their articular surface, but only at certain points; for instance, around the central ligaments which become converted into bones, and around the margins, especially the posterior margin of the bones. This leaves a relatively large surface where no union has occurred between the bones, yet the joint is obliterated. The margins of the bone taking part in the process are interesting, the ligamentous material immediately covering, say, the magnum and medium is gradually converted into bone, the pattern of the bony deposits corresponding very closely to the direction of the fibres and shapes of the ligaments. It is, therefore, largely due to conversion of ligaments into ossific material that the external deposit on the joint becomes apparent. The most uncommon part to be involved is the joint between the calcis and astragalus; the most serious the joint between the astragalus and magnum and that between the head of the metatarsal and medium.

What is the best way of judging a hock, and what are the fallacies to avoid?

Writer's views: Hocks should be judged by sight and touch, and one hock should always be compared with its fellow.

In judging by sight the observer must look at both hocks from the same stand-point. If the hock-joint was cylindrical in shape it would not matter what position we occupied in comparing the two; but inasmuch as there are a considerable number of different surfaces on the joint, if we want to compare identical areas with each other we must insure that the position we occupy in each case will admit of this. Get the hind-feet together if possible, or at any rate in the same plane; unless this simple precaution be adopted considerable error will arise.

Standing in front of the horse and looking between the fore-legs an immediate comparison may be made of the inner contour of both joints. Standing to the side of the horse and still to the front, somewhat in a line with the shoulder, the next contour of the hock is inspected and at once compared with its fellow as seen from the opposite side of the body. In both cases identical parts of the joint must be looked at, and it is a good rule to draw mentally an imaginary line down the front of the hock-joint so as to divide it into two equal parts, and to stand opposite this vertical line. In this way we can insure viewing both hocks from the same stand-point. Visual observations should invariably be repeated.

Enlargements seen from the front on viewing between the fore-legs are often situated far back on the joint, involving the head of the inner splint-bone; if this really be the case a view from the back will at once confirm the opinion. Enlargements seen when viewed from the shoulder position are toward the anterior part of the joint. In viewing from the front I never stand close to the horse, but at least nine feet, and often farther, away from the fore-legs.

To view a hock is a simple matter with practice; to handle it is always difficult. The reason of this is that to compare two hocks from the same side of the body different fingers are employed in passing over identical regions. For instance, standing on the near side, and feeling the near hock, the index-finger of the right hand is to the front, while if we feel the off hock from the near side the index-finger is to the rear of the joint; in other words, corresponding parts of the joints are examined with different fingers. This error may be corrected by examining the near hock with the right hand, and the off one with the left; but this introduces another difficulty—we have mentally to transfer to the left hand the impression conveyed by the right, and the transferrence is an extremely difficult matter where only a slight difference in the size of the joints exists. No doubt the two factors I have named constitute the difficulty attending an examination of the hock by the sense of touch.

An examination of the hock by touch can only be carried out with assurance when we can recognize with our eyes shut the structures beneath the finger; a careful and accurate knowledge of surface anatomy is, therefore, required, and is of inestimable value. Unfortunately, the greater part of the bones of the hock are clothed in ligamentous tissue, through which it is practically impossible to feel; the only region where little but skin exists between the examiner's finger and the bones of the joint is a small part immediately behind the saphena vein; here we can feel the head of the large metatarsal, a portion of the head of the small metatarsal, and a piece including the ridge on the cuneiform medium; we cannot feel the magnum nor the parvum, both are covered by the internal slip of the flexor metatarsi tendon.

So much for the parts posterior to the saphena vein. Anterior to this landmark a portion of the medium can be felt in well-bred horses; the head of the large metatarsal can also be felt, but the magnum cannot be examined, owing to the ligamentous structures there situated. In this way an examination of the structures in the front and inner aspect of the hock-joint loses much of its value, owing to the impossibility of feeling the part distinctly through the ligamentous tissue. When, however, marked enlargements occur, such can be detected whether they be anterior or posterior to the saphena vein. A greatly distended saphena will give an undue prominence to the hock; but this error is readily corrected by the sense of touch.

The inner profile of hocks free from spavin is not the same in all horses. This is due to anatomical peculiarities. The bones of the hock may be unduly prominent, especially the head of the metatarsal bone, the ridge on the medium, and the head of the inner splint-bone. To such hocks the term "coarse" is legitimately applied; one hock, however, cannot be "coarse" while its fellow is "fine;" they must both be coarse, or both be fine, to constitute a normal condition; hence the rule of practice that the hocks should be pairs. Experience shows that an enlargement which constitutes a spavin in one horse may in another be perfectly normal, and it is this paradox which expresses in a nutshell the difficulties of hock-judging.

Finally, never give an opinion about a doubtful hock unless it be examined from both sides of the body. Cases are frequently met with where an enlargement, say on the near hock, can be felt when the joint is examined from the near side, while from the opposite side of the body the enlargement on the near hock can no longer be detected, but the off joint now appears larger than its fellow. Such cases are due to the error on the part of the examiner, and may generally be proved to be so by visual inspection.

What are the conditions which arouse suspicion as to the soundness of a hock?

Writer's view: I only know of two, viz., a prominent ridge on the medium and a prominent head on the large or small metatarsal bone. The only method I know of to prevent falling into an error is the careful comparison of both joints; if they are both the same shape and size, it is fair to assume the prominence is normal. It is in such cases that a skiagraph of the hock would be most valuable, but at present the application of the X-rays is not practicable.

Will the spavin lame or not?

Writer's view: This is a very practical and important question, to which my answer to an inquirer is, "I don't know." At the same time, as we know more about these matters than our clients, we may be prepared to advise them without committing ourselves to any positive statement, and in so doing we should consider the age of the animal, the nature of the work it is called upon to perform, and the position of the enlargement. On the question of age, a young horse is, I think, more likely to go lame from spavin than an old one; on the other hand, recovery from the same is more likely than in the old horse. I think it will be found that ankylosis is the most common termination in the young, while articular disease is probably more frequent in the aged. Spavins far forward on the joint are more likely to be troublesome than enlargements far back, as they are more under the line of weight; but, as I have previously mentioned, in all probability the majority of enlargements far back are not spavins, but only well-developed heads to the splint-bones. A horse may have a spavin develop without any lameness being produced, and I have even seen articular disease between the calcis and astragalus without lameness.

Spavin may be hereditary, and I have often asked myself whether hereditary spavins give the least trouble or no trouble at all, while the non-hereditary ones are serious causes of unsoundness?

What is the prognosis of spavin?

Writer's view: As a rule, good; but again the factors of age and the position of the enlargement play a most important part in the ultimate issue. An enlargement high up on the hock, viz., on the projection of the astragalus, I look upon in the most serious light. I think they are invariably articular. The same applies to spavin low down.

Persistent lameness unrelieved by exercise is a bad symptom; such cases are frequently articular. Persistent lameness with no enlargement of the joint is an extremely serious symptom; it points to articular trouble. Within the bounds of moderation a large spavin is better than a small one, as the latter is often associated with articular disease.

What line of treatment is the best to adopt?

Writer's view: Nothing to add to what is already well known. —*Veterinary Record*, August 26, 1899.

